# A Fast Clustering Algorithm for Data with a Few Labeled Instances

**DOI:** 10.1155/2015/196098

**Published:** 2015-03-11

**Authors:** Jinfeng Yang, Yong Xiao, Jiabing Wang, Qianli Ma, Yanhua Shen

**Affiliations:** ^1^Electric Power Research Institute of Guangdong Power Grid Corporation, Guangzhou 510080, China; ^2^School of Computer Science and Engineering, South China University of Technology, Guangzhou 510006, China; ^3^School of Materials Science and Engineering, South China University of Technology, Guangzhou 510006, China

## Abstract

The diameter of a cluster is the maximum intracluster distance between pairs of instances within the same cluster, and the split of a cluster is the minimum distance between instances within the cluster and instances outside the cluster. Given a few labeled instances, this paper includes two aspects. First, we present a simple and fast clustering algorithm with the following property: if the ratio of the minimum split to the maximum diameter (RSD) of the optimal solution is greater than one, the algorithm returns optimal solutions for three clustering criteria. Second, we study the metric learning problem: learn a distance metric to make the RSD as large as possible. Compared with existing metric learning algorithms, one of our metric learning algorithms is computationally efficient: it is a linear programming model rather than a semidefinite programming model used by most of existing algorithms. We demonstrate empirically that the supervision and the learned metric can improve the clustering quality.

## 1. Introduction

Clustering is the unsupervised classification of instances into clusters in a way that attempts to minimize the intracluster distance and to maximize the intercluster distance. Two criteria commonly used to measure the quality of a clustering are diameter and split. The diameter of a cluster is the maximum distance between pairs of instances within the same cluster, and the split of a cluster is the minimum distance between instances within the cluster and instances outside the cluster. Clearly, the diameter of a cluster is a natural indication of homogeneity of the cluster and the split of a cluster is a natural indication of separation between the cluster and other clusters.

Many authors studied optimization problems related to the diameter or the split of cluster, for example, to minimize the maximum cluster diameter [1–4]; minimize the sum of cluster diameters or radii [5–8]; or maximize the ratio of the minimum split to the maximum diameter [9]. The well-known single-linkage clustering and the complete-linkage clustering also optimize the two criteria, respectively: the former maximizes the minimum cluster split, and the later attempts to minimize the maximum cluster diameter.

Ackerman and Ben-David [10] defined a set of axioms that a measure of cluster-quality should satisfy* scale invariance*,* isomorphism invariance*,* weak local consistency*, and* cofinal richness*, and they showed that the RSD clustering criterion, that is, maximizing of the ratio of the minimum split to the maximum diameter, satisfies those axioms. Given data *X*, let RSD_opt_(*X*) be the maximum RSD of *X* among all possible partitions of *X* into *k* clusters. If RSD_opt_(*X*) > 1, the optimal solution with respect to the RSD criterion has the following property: the distance between each pair of instances in different clusters is larger than that of each pair of instances within the same cluster. Hence, we say that data *X* is* well-clusterable* if RSD_opt_(*X*) > 1, and *X*′ are* more clusterable* than *X* if RSD_opt_(*X*′) > RSD_opt_(*X*).

Ackerman and Ben-David [11] showed that if RSD_opt_(*X*) > 1, then the optimal solution with respect to the RSD criterion can be found in time *O*(*n* ^2^log⁡*n*), where *n* is the number of instances in *X*. In this paper, we further show that if RSD_opt_(*X*) > 1, then the optimal solution with the following criteria can be found using Gonzalez's algorithm [1] in linear time: maximizing RSD, maximizing the minimum split, and minimizing the maximum diameter.

However, the condition of RSD_opt_(*X*) > 1 is too strong and unrealistic for real world data. So, a natural problem arises if *X* is poorly clusterable (RSD_opt_(*X*) ≪ 1), whether *X* can be made more clusterable by a metric learning approach and thus Gonzalez's algorithm together with the learned metric can perform better than together with the original metric.

In the clustering literature, there are commonly two methods to add supervision information into clustering. First, adding a small portion of the training data into unlabeled data, this method is also called semisupervised learning [12, 13]. Second, instead of specifying the class labels, pairwise constraints are specified [14, 15]: a pairwise must-link constraint corresponds to the requirement that the involved two instances must be within the same cluster, whereas the two instances involved in a cannot-link constraint must be in different clusters.

Metric learning can be grouped into two categories, that is, unsupervised and supervised metric learning. In this paper, we focus on supervised metric learning. Supervised metric learning attempts to learn distance metrics that keep instances with the same class label (or with a must-link constraint) close and separate instances with different class labels (or with a cannot-link constraint) far away. Since there are many possible ways to realize this intuition, a great number of algorithms have been developed for supervised metric learning, for example, Local Linear Discriminative Analysis (LLDA) [16], Relevant Components Analysis (RCA) [17], Xing et al.'s algorithm [18], Locally Linear Metric Adaptation (LLMA) [19], Neighborhood Component Analysis (NCA) [20], Discriminative Component Analysis (DCA) [21], Local Fisher Discriminant Analysis (LFDA) [22], Large Margin Nearest Neighbor (LMNN) [23], Local Distance Metric (LDM) [24], Information-Theoretic Metric Learning (ITML) [25], Laplacian Regularized Metric Learning (LRML) [26], Generalized Sparse Metric Learning (GSML) [27], Sparse Distance Metric Learning (SDML) [28], Multi-Instance MEtric Learning (MIMEL) [29], online-reg [30], Constrained Metric Learning (CML) [31], mixture of sparse Neighborhood Components Analysis (msNCA) [32], Metric Learning with Multiple Kernel Learning (ML-MKL) [33], Least Squared residual Metric Learning (LSML) [34], and Distance Metric Learning with eigenvalue (DML-eig) [35].

Overall, empirical studies showed that supervised metric learning algorithms can usually outperform unsupervised ones by exploiting either the label information or the side information presented in pairwise constraints. However, despite extensive studies, most of the existing algorithms for metric learning have one of the following drawbacks: it needs to solve a nontrivial optimization problem, for example, a semidefinite programming problem, there are parameters to tune, and the solution is local optimal.

In this paper, we present two simple metric learning models to make data more clusterable. The two models are computationally efficient, parameter-free, and local-optimality-free. The rest of this paper is organized as follows.  Section 2 gives some notations and the definitions of clustering criteria used in the paper.  Section 3 gives Gonzalez's farthest-point clustering algorithm for unsupervised learning, presents a nearest neighbor-based clustering algorithm for the semi-supervised learning, and discusses the properties of the two algorithms. In Section 4, we formularize the problem of making data more clusterable as a convex optimization problem.  Section 5 presents the experimental results. We conclude the paper in Section 6.

## 2. Notations and Preliminary

We use the following notations in the rest of the paper.

 |·|: the cardinality of a set.
 
*X* ⊂ ℜ^*d*^: the set of instances (in *d*-dimension space) to be clustered.
 
*d*(*x*, *y*): the Euclidian distance between *x* ∈ *X* and *y* ∈ *X*.
 
*S*_1_, *S*_2_,…, *S*_*k*_: the *k* small subsets of *X* with given labels, that is, the supervision. In this paper, we assume that either *S*_*i*_ ≠ Φ for *i* = 1,2,…, *k* (the case of semisupervised learning) or *S*_*i*_ = Φ for *i* = 1,2,…, *k* (the case of unsupervised learning).
 
*℘*: the set of all partitions of *n* objects into *k* nonempty and disjoint clusters {*C*_1_, *C*_2_,…, *C*_*k*_}.

**Definition 1 .**

Given *S*_1_, *S*_2_,…, *S*_*k*_, we say that a partition *P* ∈ *℘* respects the semi-supervised constraints if *P* satisfies the following conditions.

1. All instances in *S*_*i*_ must be within the same cluster of *P* for *i* = 1,2,…, *k*, and
2. Any pair of instances *x* ∈ *S*_*i*_ and *y* ∈ *S*_*j*_, *x* and *y* must be in different clusters of *P* for *i*, *j* = 1,2,…, *k*, and *i* ≠ *j*.

In the rest of the paper, we use *℘*_ssc_ to denote the subset of *℘* that respects the semisupervised constraints, and we require that any partition in the context of semisupervised learning should respect the semisupervised constraints.

**Definition 2 .**

For a set *C* of objects, the split *s*(*C*) of *C* is defined as

(1) $$\begin{array}{c} s\left(C\right)=\underset{x\in C,y\notin C}{\min}d\left(x,y\right). \end{array}$$

For a partition *P* = {*C*_1_, *C*_2_,…, *C*_*k*_} ∈ *℘*, the split *s*(*P*) of *P* is the minimum *s*(*C*_*i*_) among *i* = 1,2,…, *k*.

**Definition 3 .**

For a set *C* of objects, the diameter *d*(*C*) of *C* is defined as

(2) $$\begin{array}{c} d\left(C\right)=\underset{x,y\in C}{\max}d\left(x,y\right). \end{array}$$

For a partition *P* = {*C*_1_, *C*_2_,…, *C*_*k*_} ∈ *℘*, the diameter *d*(*P*) of *P* is the maximum diameter *d*(*C*_*i*_) of *C*_*i*_ among *i* = 1,2,…, *k*.

**Definition 4 .**

The unsupervised and semisupervised max-min split problems are defined as, respectively,

(3) $$\begin{array}{c} \underset{P\in ℘}{\max}\;s\left(P\right), \end{array}$$

(4) $$\begin{array}{c} \underset{P\in {℘}_{\text{scc}}}{\max}s\left(P\right). \end{array}$$

**Definition 5 .**

The unsupervised and semisupervised min-max diameter problems are defined as, respectively,

(5) $$\begin{array}{c} \underset{P\in ℘}{\min}\;d\left(P\right), \end{array}$$

(6) $$\begin{array}{c} \underset{P\in {℘}_{\text{scc}}}{\min}d\left(P\right). \end{array}$$

**Definition 6 .**

The unsupervised and semisupervised max-RSD problems are defined as, respectively,

(7) $$\begin{array}{c} \underset{P\in ℘}{\max}\frac{s\left(P\right)}{d\left(P\right)}, \end{array}$$

(8) $$\begin{array}{c} \underset{P\in {℘}_{\text{scc}}}{\max}\frac{s\left(P\right)}{d\left(P\right)}. \end{array}$$

For the unsupervised max-RSD problem, Wang and Chen [9] presented an exact algorithm for *k* = 2 and a 2-approximation algorithm for *k* ≥ 3; however the worst-case time complexity of both algorithms is *O*(*n* ^3^) and thus impractical for large-scale data.

Let *S*⊆*X*; we use *dMax*⁡(*x*, *S*) to denote the maximum distance between the instance *x* and instances in *S*; that is, *dMax*⁡(*x*, *S*) = max⁡{*d*(*x*, *y*)∣*y* ∈ *S*}; similarly, *dMin*⁡(*x*, *S*) = min⁡{*d*(*x*, *y*)∣*y* ∈ *S*}.

## 3. Well-Clusterable Data: Find the Optimal Solution Efficiently

In this section, we show that if RSD_opt_(*X*) > 1, the max-RSD problem, the max-min split problem, and the min-max diameter problem can be simultaneously solved by Gonzalez's algorithm for unsupervised learning in Section 3.1 and by a nearest neighbor-based algorithm for semisupervised learning in Section 3.2, respectively. At the same time, we also discuss the properties of the two algorithms for the case of RSD_opt_(*X*) ≤ 1.

### 3.1. Unsupervised Learning

The farthest-point clustering (*FPC*) algorithm proposed by Gonzalez [1] is shown in Algorithm 1, where the meaning of nearest neighbor is its literal one as (9); that is, *p*'s nearest neighbor in *R* is *q*,

(9) $$\begin{array}{c} q=\underset{u\in R}{\arg\;\min}\;d\left(p,u\right). \end{array}$$

**Theorem 7 .**

For unsupervised learning, if *RSD*_*opt*_(*X*) > 1, then the partition *P* returned by* FPC* is simultaneously the optimal solution of the max-RSD problem, the max-min split problem, and the min-max diameter problem.

**Proof**

(a) The proof of the max-RSD problem: let *P*′ = {*C*_1_, *C*_2_,…, *C*_*k*_} be the optimal partition of the max-RSD problem; then RSD(*P*′) > 1, and we have

(10) $$\begin{array}{c} \forall p,q\in C_{i},\;\;\forall u\notin C_{i}\text{:}\;d\left(p,q\right)<d\left(p,u\right)\;\forall i. \end{array}$$

We prove the following proposition: any pair of instances in *R* (see Algorithm 1) must be in different clusters of *P*′; that is, *R* contains exactly one instance of each cluster *C*_*i*_,  *i* = 1,2,…, *k*. If this holds, then by (10), for any instance *q* ∈ *C*_*i*_, *i* = 1,2,…, *k*, its nearest neighbor in *R* must be the instance *p* such that *p* also belongs to *C*_*i*_, and hence *P* = *P*′.

We prove the proposition by contradiction. Assume that there exists a pair of instances *p* and *q* in *R* so that they belong to the same cluster *C*_*r*_ for some *r*. Without loss of generality, let *p* be selected into *R* before *q*. Then *dMin*⁡(*q*, *R*) ≤ *d*(*q*, *p*) when selecting *q* into *R*. Note that |*R* | <*k* before selecting *q*; there exists at least one cluster *C*_*t*_  (*t* ≠ *r*) such that no instance in *C*_*t*_ belongs to *R*. By (10), for any *q*′ ∈ *C*_*t*_, we have *dMin*⁡(*q*′, *R*) > *d*(*q*, *p*) ≥ *dMin*⁡(*q*, *R*); *q* has no chance to be selected into *R* since we should select the instance *q*′ with the maximum *dMin*⁡(*q*′, *R*), and thus the proposition holds.

(b) Since separating any pair *p*, *q* of instances within the same cluster of *P* into different clusters will strictly decrease the split of the resulted partition, the conclusion for the max-min split problem holds.

(c) Since grouping any pair *p*, *q* of instances in different clusters of *P* into the same cluster will strictly increase the diameter of the resulting partition, the conclusion for the min-max diameter problem holds.

Clearly, the time complexity of *FPC* is *O*(*nk*) by maintaining a nearest neighbor table that records the nearest neighbor in *R* of each instance *p* ∈ *X* − *R* and the corresponding distance between *p* and its nearest neighbor in *R*. The space complexity is *O*(*n*). So, the time complexity and the space complexity are both linear with *n* for a fixed *k*. Using a more complicated approach, the *FPC* algorithm can be implemented in *O*(*n*log⁡*k*), but the implementation was exponentially dependent on the dimension *d* [3].

Now, a natural problem arises: if RSD_opt_(*X*) ≤ 1, how does the* FPC* algorithm perform? Although, in this paper, we cannot give performance guarantee of the* FPC* algorithm for the max-RSD problem and the max-min split problem if RSD_opt_(*X*) ≤ 1, Gonzalez [1] proved the following theorem (see also [2, 3]).

**Theorem 8 (see [1]).**

The* FPC* is a 2-approximation algorithm for the unsupervised min-max diameter problem with the triangle inequality satisfied for any *k*. Furthermore, for *k* ≥ 3, the (2 − *ε*)-approximation of the unsupervised min-max diameter problem with the triangle inequality satisfied is NP-complete for any *ε* > 0.

So as far as the approximation ratio is concerned, the* FPC* algorithm is the best for the unsupervised min-max diameter problem unless P = NP.

### 3.2. Semi-Supervised Learning

For semisupervised learning, we present a nearest neighbor-based clustering (*NNC*) algorithm as shown in Algorithm 2. The algorithm is self-explanatory, and we do not give a further explanation.

**Theorem 9 .**

For semiunsupervised learning, if *RSD*_*opt*_(*X*) > 1, then the partition *P* returned by* NNC* is simultaneously the optimal solution of the semisupervised max-RSD problem, the semisupervised max-min split problem, and the semisupervised min-max diameter problem.

**Proof**

The proof of max-RSD(*P*) problem: let *P*′ = {*C*_1_′, *C*_2_′,…, *C*_*k*_′} be the optimal partition of the semisupervised max-RSD problem. Since *P*′ respects the supervision, we can replace *S*_*i*_ by a super-instance *α*_*i*_ for *i* = 1,2,…, *k*; then each cluster *C*_*i*_′ contains exactly one super-instance *α*_*i*_ for *i* = 1,2,…, *k* (without loss of generality, here we assume that *α*_*i*_ is in the cluster *C*_*i*_′ for *i* = 1,2,…, *k*). Let *P* = {*C*_1_, *C*_2_,…, *C*_*k*_}; then according to the algorithm* NNC*, each cluster also contains exactly one super-instance, and without loss of generality, we also assume that *α*_*i*_ is in the cluster *C*_*i*_ for *i* = 1,2,…, *k*. For each unlabeled instance *p* ∈ *C*_*r*_′ for *r* = 1,2,…, *k*, since RSD_opt_(*X*) > 1, we have *d*(*p*, *α*_*r*_) = *dMax*⁡(*p*, *S*_*r*_) < *d*(*p*, *α*_*i*_) = *dMax*⁡(*p*, *S*_*i*_) for any *i* ≠ *r*, and the nearest neighbor of *p* in {*α*_1_, *α*_2_,…, *α*_*k*_} is *α*_*r*_, so *C*_*r*_′ = *C*_*r*_ for *r* = 1,2,…, *k*, and thus *P*′ = *P*.

The proofs for the semisupervised max-min split problem and the semisupervised min-max problem are similar to (b) and (c) in the proof of Theorem 7 respectively, and here we omit it.

The time complexity of* NNC* using a simple implementation is

(11) $$\begin{array}{c} \overset{k}{\underset{i=1}{\sum }}O\left(n\left|S_{i}\right|\right)+O\left(nk\right). \end{array}$$

The space complexity of* NNC* is *O*(*n*). Since we assume that *S*_*i*_ are small sets for *i* = 1,2,…, *k*, the time and space complexities are also linear with *n* when |*S*_*i*_| are regarded as constants for *i* = 1,2,…, *k*.

Similar to Theorem 8, we have the following theorem for the semisupervised min-max diameter problem.

**Theorem 10 .**

*NNC* is a 2-approximation algorithm for the semisupervised min-max diameter problem with the triangle inequality satisfied.

**Proof**

Let *S* = {*α*_1_, *α*_2_,…, *α*_*k*_} (see the proof of Theorem 9), *δ* = max⁡{*d*(*S*_1_), *d*(*S*_2_),…, *d*(*S*_*k*_)}, and *σ* = max⁡{*dMin*⁡(*q*, *S*)∣*q* is an unlabelled instance} and let *p* be any unlabelled instance such that *dMin*⁡(*p*, *S*) = *σ*. Since the optimal partition of a semisupervised min-max diameter problem must respect the supervision, we have *d*_opt_(*X*) ≥ *δ*, where *d*_opt_(*X*) denotes the diameter of the optimal solution of the semisupervised min-max diameter problem; at the same time, *p* and *α*_*i*_ for some *i* ∈ {1,2,…, *k*} must be within the same cluster of the optimal solution, so *d*_opt_(*X*) ≥ *σ*; therefore *d*_opt_(*X*) ≥ max⁡{*δ*, *σ*}. Now consider the partition *P* = {*C*_1_, *C*_2_,…, *C*_*k*_} returned by* NNC*. Since each unlabeled instance *q* is assigned into its nearest neighbor in *S*, so, for any cluster *C*_*i*_ of *P* for *i* = 1,2,…, *k* (assume that the super-instance in *C*_*i*_ is *α*_*i*_), we have *d*(*q*, *α*_*i*_) ≤ *σ*, and *d*(*C*_*i*_) ≤ 2*σ* by the triangle equality. So, *d*(*P*) ≤ max⁡{2*σ*, *δ*} ≤ 2*d*_opt_(*X*), and the theorem holds.

## 4. The Metric Learning Models

If the given data are poorly clusterable, that is, the RSD_opt_(*X*) is far less than one, the algorithms* FPC* and* NNC* may perform poorly. Given the supervision, we use metric learning to make the supervised data more clusterable, and then the two algorithms can be used with the new metric.

Supervised metric learning attempts to learn distance metrics that keep instances with the same class labels (or with a must-link constraint) close and separate instances with different class labels (or with a cannot-link constraint) far away. As discussed in the first section, there are many possible ways to realize this intuition; for example, Xing et al. [18] presented the following model:

(12)  min⁡M ∑(x,y)∈Sx−yM2

(13)  s.t.  ∑(x,y)∈Dx−yM≥1

(14)     M⪰0.

In the above model, *S* denotes the set of must-link constraints, *D* denotes the set of cannot-link constraints, *M* is a *d* × *d* Mahalanobis distances matrix, and ‖*x* − *y*‖_*M*_ denotes the distance *d*(*x*, *y*) between two instances *x* and *y* ∈ *X*⊆ℜ^*d*^ with respect to *M*; that is,

(15) $$\begin{array}{c} {\left\|x-y\right\|}_{M}=\sqrt{{\left(x-y\right)}^{T}M\left(x-y\right)}, \end{array}$$

where *T* denotes the transpose of a matrix or a vector. The constraint (14) requires that *M* should be a positive semidefinite matrix; that is, ∀*x* ∈ ℜ^*d*^, *x* ^*T*^ *Mx* ≥ 0. The choice of the constant 1 on the right hand side of (13) is arbitrary but not important, and changing it to any other positive constant *c* results only in *M* being replaced by *c* ^2^ *M*.

Note that the matrix *M* can be either a full matrix or a diagonal matrix. In natural language, Xing et al.'s model minimizes the sum of the square of distance with respect to *M* between pairs of instances with must-link constraints subject to the following constraints: (a) the sum of distances with respect to *M* between pairs of instances with cannot-link constraints is greater than or equal to one, and (b) *M* is a positive semidefinite matrix.

Xing et al.'s model, as well as most of the existing metric learning, is a semidefinite programming problem and thus computationally expensive and even intractable in high dimensional space for the case of full matrix.

Inspired by the RSD clustering criterion, we propose two metric learning models: one learns a full matrix and the other learns a diagonal matrix. In this section, the supervision can be given either in the form of labeled sets *S*_1_, *S*_2_,…, *S*_*k*_ or in the form of pairwise constraints.

### 4.1. The Labeled Sets

Given the supervision *S*_1_, *S*_2_,…, *S*_*k*_, we want to learn a Mahalanobis distances matrix *M* such that the minimum split with respect to *M* among *S*_*i*_,  *i* = 1,2,…, *k*, is maximized subject to the following constraints: (a) the distance between each pair of instances with the same class label is less than or equal to one and (b) *M* is a positive semidefinite matrix. Formally, we have the following optimization problem (the case of full matrix).

*The Case of Full Matrix.* Consider

(16)  max⁡M s

(17)   s.t.  ∀x∈Si,  y∈Sj: x−yM≥s,      hii,j=1,2,…,k, i≠j

(18)        ∀x,y∈Si: x−yM≤1, i=1,2,…,k

(19)        M⪰0

(20)        s≥0.

The constraint (17) requires that the scalar variable *s* (the minimum split) is the minimum among distances between pairs of instances with different class labels. The constraint (18) requires that the distance between each pair of instances with the same class label is less than or equal to one. The optimization objective is to maximize *s*. Similar to (13), the choice of the constant 1 on the right hand side of (18) is arbitrary but not important and can be set to any positive constant.

The full matrix model is a SDP optimization problem, and, theoretically, the global optimal solution can be solved efficiently [36]. However, when *M* is a full matrix, the number of variables (|*M*|) is quadratic in *d*, and thus it is prohibitive for problems with a large number of dimensions. To avoid this problem, we can require that *M* is a diagonal matrix. Since *M* is a diagonal matrix, *M* is a positive semidefinite matrix if and only if *M*_*ii*_ ≥ 0 for *i* = 1,2,…, *d*, where *M*_*ii*_ is the *i*th diagonal entry. So, learning a diagonal matrix *M* is equivalent to learning a vector *z* ∈ ℜ^*d*^ using the following model (the case of diagonal matrix). 

*The Case of Diagonal Matrix.* Consider

(21)  max⁡z s

(22)   s.t.  ∀x∈Si,  y∈Sj: x−yz≥s,        i,j=1,2,…,k, i≠j

(23)       ∀x,y∈Si: x−yz≤1, i=1,2,…,k

(24)       z≥0

(25)     s≥0,

where

(26) $$\begin{array}{c} {\left\|x-y\right\|}_{z}=\sqrt{\overset{d}{\underset{i=1}{\sum }}z_{i}{\left(x_{i}-y_{i}\right)}^{2}}. \end{array}$$

The constraint (24) requires that each component of *z* should be greater than or equal to zero.

Now since the optimization objective and all constraints are linear, the above optimization problem is a linear programming problem with *d* + 1 variables, and *k* × |*S*_*i*_ | ×(|*S*_*i*_ | −1)/2 + *k* × (*k* − 1) × |*S*_*i*_|^2^/2 + (*d* + 1) inequality constraints (assume that *S*_*i*_ has equal size). When |*S*_*i*_| is small for *i* = 1,2,…, *k*, the global optimal solution can be efficiently found using some optimization tool package, for example, the MATLAB* linprog* function, or the CVX—MATLAB software for disciplined convex programming (http://cvxr.com/cvx/download/).

### 4.2. Pairwise Constraints

If the supervision is given in the form of pairwise constraints, that is, the* must*-*link* and* cannot*-*link* constraints, the models also work after a minor modification. Let ML be the set of must-link constraints, and let CL be the set of cannot-link constraints; then the full matrix model and the diagonal matrix model should be modified as follows: substituting 17′ for (17), 18′ for (18), 22′ for (22), and 23′ for (23), respectively,

$$\begin{array}{cc} \left(17^{\prime }\right) & \forall \left(x,y\right)\in \text{ML:}\;{\left\|x-y\right\|}_{M}\leq 1, \end{array}$$

$$\begin{array}{cc} \left(18^{\prime }\right) & \forall \left(x,y\right)\in \text{CL:}\;{\left\|x-y\right\|}_{M}\geq s, \end{array}$$

$$\begin{array}{cc} \left(22^{\prime }\right) & \forall \left(x,y\right)\in \text{ML:}\;{\left\|x-y\right\|}_{z}\leq 1, \end{array}$$

$$\begin{array}{cc} \left(23^{\prime }\right) & \forall \left(x,y\right)\in \text{CL:}\;{\left\|x-y\right\|}_{z}\geq s. \end{array}$$

However, if the supervision is given in the form of pairwise constraints, it is nontrivial to decide whether there is a partition *P* of *X* such that *P* satisfies all of those pairwise constraints (and we call it the feasibility problem). For CL constraints, Davidson and Ravi showed that the feasibility problem is equivalent to the *k*-colorability problem [37] and thus NP-complete [38], whereas the feasibility problem is trivial if the supervision is given in the form of labeled sets. Of course, if we do not require that all of those pairwise constraints should be satisfied, the* FPC* algorithm can be naturally used together with the metric learned from the pairwise constraints.

Clearly, the metric learning models proposed in this paper are practicable only when the cardinality of sets of labeled instances or the number of pairwise constraints is small. Otherwise, the problem is usually overconstrained and there is no feasible solution.

## 5. The Experimental Results

### 5.1. The Compared Algorithms and Benchmark Datasets

To validate whether semisupervised learning performs better than unsupervised one, whether metric learning can improve clustering quality, and whether our metric learning model performs better than Xing et al.'s one for the *FPC* and *NNC* algorithms, we implemented the following algorithms:

- the* FPC* algorithm as shown in Algorithm 1;
- the* NNC* algorithm as shown in Algorithm 2;
- the* FPC* with our metric learning model (the case of diagonal matrix) (*FPC*_*Diag*); that is, we first use our metric learning model to learn a vector *z* and then use the* FPC* clustering algorithm with the learned vector; that is, the distance is computed using (26);
- the* NNC* with our metric learning model (the case of diagonal matrix) (*NNC*_*Diag*);
- the* FPC* with Xing et al.'s metric learning algorithm (also using the diagonal matrix) (*FPC*_*Xing*); that is, we first use Xing et al.'s metric learning algorithm to learn a vector *z* and then use the* FPC* clustering algorithm with the learned vector;
- the* NNC* with Xing et al.'s metric learning algorithm (also using the diagonal matrix) (*NNC*_*Xing*).

We also implemented the following algorithms as baseline approaches. The reason that we select *k-means* to compare is that *k-means* is very simple and also a linear time algorithm when regarding *k* and the repetition times as constants:

- *the constrained k-means* [39] with Xing et al.'s metric learning algorithm (*CopK*_*Xing*);
- *pairwise constrained k-means* with Xing et al.'s metric learning algorithm (*PCK*_*Xing*) [40, 41].

For Xing et al.'s metric learning method, the code is downloaded from Xing's home page: http://www.cs.cmu.edu/~epxing/publications.html.

We conduct experiments on twenty UCI real world datasets obtained from the Machine Learning Repository of the University of California, Irvine [42]. The information about those datasets is summarized in Table 1.

### 5.2. The Experiments Setup

We first make the following preprocessing: for a nominal attribute with *I* different values, we replace these values by *I* integers 1,2,…, *I*, and then all attributes are normalized to the interval [1, 2].

Except* Ecoli*, |*S*_*i*_| is set to five for *i* = 1,2,…, *k*. Because the smallest number of instances is two among eight classes in the dataset* Ecoli*, |*S*_*i*_| is set to two for *i* = 1,2,…, *k*.

Xing et al.'s metric learning is carried out on the original pairwise constraints: ML = {(*p*, *q*)∣*p*, *q* ∈ *S*_*i*_, *i* = 1,2,…, *k*} and CL = {(*p*, *q*)∣*p* ∈ *S*_*i*_, *q* ∈ *S*_*j*_, *i*, *j* = 1,2,…, *k*, *i* ≠ *j*}. In the phase of clustering for* CopK*_*Xing* and* PCK*_*Xing*, it is the centroid *c*_*i*_ of *S*_*i*_ that participates in the clustering process, which guarantees that all must-link constraints are satisfied.

The stop condition is either the repetition times are more than 100 or the objective difference between two consecutive repetitions is less than 10^−6^.

We use the Rand Index [43] to measure the clustering quality in our experiments. The Rand Index reflects the agreement of the clustering result with the ground truth. Here, the ground truth is given by the data's class labels. Let *n*_*s*_ be the number of instance pairs that are assigned to the same cluster and have the same class label, and let *n*_*d*_ be the number of instance pairs that are assigned to different clusters and have different class labels. Then, the Rand Index is defined as

(27) $$\begin{array}{c} \text{RI}=\frac{2\left(n_{s}+n_{d}\right)}{\left(n\left(n-1\right)\right)}. \end{array}$$

All algorithms are implemented in MATLAB R2009b, and experiments are carried out on a 2.6 GHz double-core Pentium PC with 2 G bytes of RAM.

### 5.3. The Mean Rand Index

Table 2 summarizes the mean Rand Index and the standard deviation over 20 random runs on twenty datasets, and the value with bold in each row is the highest. Table 2 shows that although no algorithm performs better than the other algorithms on all datasets, in general we can draw the following conclusion.

1. The supervision can significantly improve the clustering quality: compared with* FPC* and* FPC*_*Diag*, the mean Rand Index of* NNC* and* NNC*_*Diag* over twenty datasets increases about 27 percent and 22 percent, respectively. Note that the increment of the Rand Index that resulted from the addition of supervision itself is very small.
2. The introducing of metric learning into an existing algorithm does not always increase its performance. However, in general, the effect of our metric learning model is positive: the win/loss ratio of* FPC*_*Diag* to* FPC* is 11/3, and the win/loss ratio of* NNC*_*Diag* to* NNC* is 8/1, where* algorithm A* defeating* algorithm B* means that the Rand Index of* A* is higher at least 0.03 than that of* B* since the standard deviation is a bit large.
3. Compared with* CopK_Xing* and* PCK_Xing*,* NNC*_*Diag* performs a little better: the win/loss ratio of* NNC*_*Diag* to* CopK_Xing* is 5/3, and the win/loss ratio of* NNC*_*Diag* to* PCK_Xing* is 6/3.
4. For the* FPC* and* NNC* clustering algorithms, the proposed metric learning model is better than Xing et al.'s method, especially for* FPC*. For* FPC*, Xing et al.'s method resulted in the fact that the performance of* FPC* significantly decreased on nine datasets and the mean Rand Index of* FPC*_*Xing* even decreases about 6 percent compared with* FPC*. The win/loss ratio of* NNC*_*Diag* to* NNC*_*Xing* is 8/1. This fact seems to advise that when selecting a metric learning model for an existing clustering algorithm, the metric learning model should correspond to the clustering criterion of the clustering algorithm.

### 5.4. The Runtime

**Figure 1:**
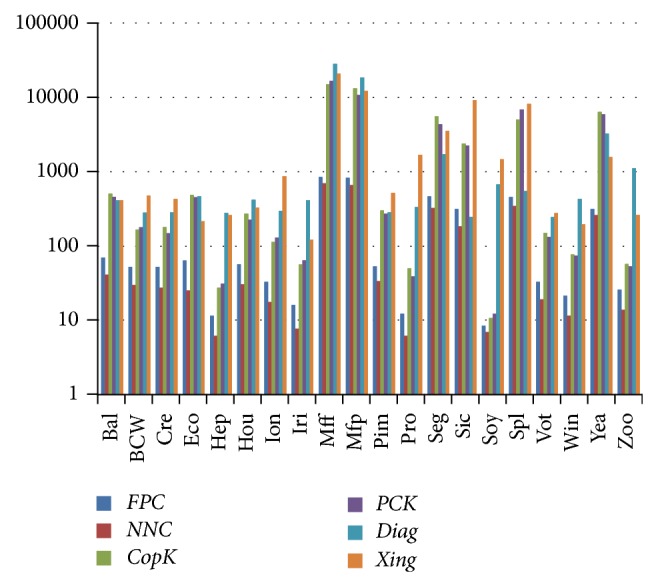
The logarithm graph of the mean runtime (milliseconds) over 20 random runs.

Figure 1 depicts the logarithm graph of the mean runtime (milliseconds) over 20 random runs, where the runtime of* FPC*,* NNC*,* CopK*, and* PCK* does not include the metric learning time. The legend* Diag* denotes the runtime of the metric learning time of our diagonal matrix model, and the legend* Xing* denotes the metric learning time of Xing et al.'s model (the diagonal matrix). So, the runtime of* FPC*_*Diag* (*NNC*_*Diag*) is the sum of the* FPC (NNC)* and the* Diag*. Similarly, the runtime of* CopK*_*Xing* (*PCK*_*Xing*) is the sum of the* CopK* (*PCK*) and the* Xing*.

Figure 1 shows that both* NNC* and* FPC* are much faster than* CopK* and* PCK*, which is consistent with their time complexities: the complexity of* FPC* and* NNC* is *O*(*nk*), whereas the complexity of* CopK* and* PCK* is *O*(*nkt*), where *t* is repetition times of *k-means*. Figure 1 also shows that Xing et al.'s model is slower than our model when the number of dimensions is relatively large, for example,* Ionosphere*,* Promoters*,* Sick*, and* Splice*. On the other hand, since the number of inequality constraints is quadratic with the number of class labels, our* Diag* model is slower than Xing et al.'s model on datasets with relatively large number of class labels, for example,* Ecoli*,* Mfeat-fac*,* Mfeat-pix*,* Yeast*, and* Zoo*.

The experimental results in Table 2 and Figure 1 show that the* FPC* algorithm is very fast, but the clustering results are unsatisfactory. The* NNC* algorithm proposed in this paper has the same time complexity as* FPC*, but the clustering quality is much more satisfactory than* FPC* if a few labeled instances are available.

## 6. Conclusion

In this paper, we studied the problem related to clusterability. We showed that if the input data are well clusterable, the optimal solutions with respect to the min-max diameter criterion, the max-min split criterion, and the max-RSD criterion can be simultaneously found in linear time for both unsupervised and semisupervised learning. For the max-RSD criterion, we also proposed two convex optimization models to make data more clusterable.

The experimental results on twenty UCI datasets demonstrate that both the supervision and the learned metric can significantly improve the clustering quality. We believe that the proposed* NNC* algorithm and metric learning models are useful when only a few labeled instances are available.

Usually, the term semisupervised learning is used to describe scenarios where both the labeled data and the unlabeled date affect the performance of a learning algorithm, which is not the case here: the supervised data is used either to induce a nearest neighbor classifier on the unlabeled data or to find a metric vector. Hence, the supervision information can be more elaborately utilized in the future.

## Figures and Tables

**Algorithm 1:**
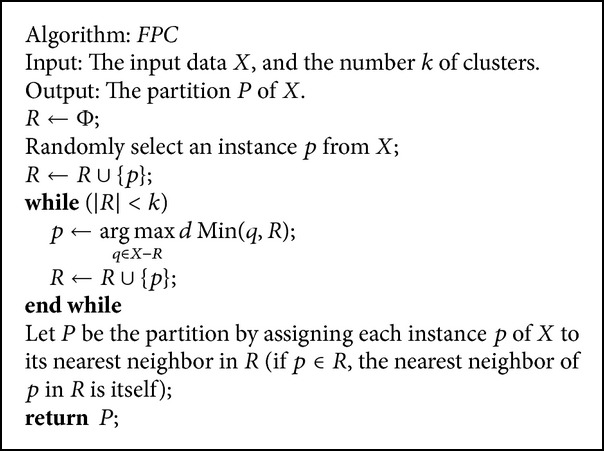
The *FPC* algorithm for unsupervised learning [1].

**Algorithm 2:**
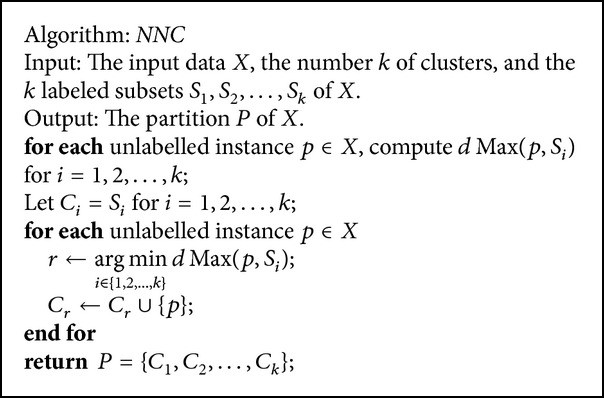
The *NNC *clustering algorithm for semisupervised learning.

**Table 1:** The information of benchmark datasets.

Dataset	Abbr.	#Class	#Attr	Size
Balance	Bal	3	4	625
Breast Cancer Wisconsin	BCW	2	9	699
Credit	Cre	2	15	653
*Ecoli*	Eco	8	7	336
Hepatitis	Hep	2	19	155
Housing	Hou	3	13	506
Ionosphere	Ion	2	34	351
Iris	Iri	3	4	150
mfeat-fac	Mff	10	216	2000
mfeat-pix	Mfp	10	240	2000
Pima	Pim	2	8	768
Promoters	Pro	2	57	106
Segmentation	Seg	7	19	2310
Sick	Sic	2	29	3772
Soybean	Soy	4	35	47
Splice	Spl	3	60	3175
Voting	Vot	2	16	435
Wine	Win	3	13	178
Yeast	Yea	10	8	1484
Zoo	Zoo	7	17	101

**Table 2:** The mean Rand Index and the standard deviation over 20 random runs (|*S*_*i*_ | = 2 for *Ecoli* and 5 for the others, *i* = 1,2,…, *k*).

Dataset	FPC	NNC	FPC_Diag	NNC_Diag	CopK_Xing	PCK_Xing	FPC_Xing	NNC_Xing
Bal	0.534 ± 0.034	0.594 ± 0.038	0.510 ± 0.033	0.588 ± 0.032	0.602 ± 0.039	0.594 ± 0.031	0.434 ± 0.002	**0.608** ± 0.067
BCW	0.629 ± 0.074	0.832 ± 0.044	0.636 ± 0.050	0.803 ± 0.023	0.840 ± 0.150	**0.860** ± 0.135	0.579 ± 0.014	0.846 ± 0.032
Cre	0.521 ± 0.013	0.601 ± 0.066	0.538 ± 0.050	0.660 ± 0.077	**0.683** ± 0.046	**0.685** ± 0.065	0.506 ± 0.006	0.625 ± 0.075
Eco	0.596 ± 0.089	**0.871** ± 0.022	0.716 ± 0.093	0.793 ± 0.021	0.816 ± 0.009	0.818 ± 0.011	0.298 ± 0.013	0.813 ± 0.033
Hep	0.599 ± 0.063	0.579 ± 0.084	0.640 ± 0.039	0.572 ± 0.063	0.566 ± 0.036	0.564 ± 0.039	**0.668** ± 0.010	0.588 ± 0.066
Hou	0.546 ± 0.023	0.603 ± 0.028	0.497 ± 0.048	**0.621** ± 0.024	0.604 ± 0.005	0.601 ± 0.006	0.467 ± 0.024	0.607 ± 0.040
Ion	0.519 ± 0.031	0.552 ± 0.023	0.549 ± 0.034	0.553 ± 0.036	0.571 ± 0.021	**0.581** ± 0.013	0.522 ± 0.013	0.543 ± 0.021
Iri	0.618 ± 0.056	0.870 ± 0.021	0.655 ± 0.025	0.907 ± 0.068	0.845 ± 0.061	0.819 ± 0.129	0.446 ± 0.096	**0.918** ± 0.026
Mff	0.691 ± 0.040	0.879 ± 0.012	0.787 ± 0.047	**0.915** ± 0.013	0.901 ± 0.014	0.903 ± 0.008	0.733 ± 0.019	0.875 ± 0.009
Mfp	0.377 ± 0.084	0.867 ± 0.013	0.795 ± 0.053	**0.906** ± 0.016	**0.906** ± 0.016	**0.909** ± 0.015	0.730 ± 0.018	0.880 ± 0.014
Pim	0.542 ± 0.014	0.544 ± 0.027	0.540 ± 0.018	0.538 ± 0.034	**0.556** ± 0.004	**0.555** ± 0.004	0.544 ± 0.001	**0.553** ± 0.024
Pro	0.502 ± 0.008	0.537 ± 0.031	0.511 ± 0.025	0.572 ± 0.061	**0.588** ± 0.063	0.579 ± 0.072	0.497 ± 0.003	0.574 ± 0.033
Seg	0.589 ± 0.103	**0.855** ± 0.015	0.397 ± 0.162	**0.854** ± 0.049	0.827 ± 0.038	0.843 ± 0.021	0.403 ± 0.099	0.821 ± 0.016
Sic	0.595 ± 0.083	0.631 ± 0.124	0.789 ± 0.114	0.835 ± 0.151	0.679 ± 0.136	0.658 ± 0.142	**0.863** ± 0.018	0.652 ± 0.095
Soy	0.669 ± 0.044	0.973 ± 0.022	0.754 ± 0.049	**0.982** ± 0.011	0.906 ± 0.080	0.843 ± 0.087	0.715 ± 0.056	0.950 ± 0.014
Spl	0.528 ± 0.005	0.516 ± 0.028	0.510 ± 0.031	0.547 ± 0.018	**0.618** ± 0.039	**0.619** ± 0.036	0.385 ± 0.000	0.539 ± 0.026
Vot	0.540 ± 0.036	0.612 ± 0.097	0.573 ± 0.074	0.769 ± 0.124	**0.773** ± 0.004	0.712 ± 0.111	0.526 ± 0.019	0.731 ± 0.071
Win	0.607 ± 0.038	0.804 ± 0.048	0.567 ± 0.076	**0.883** ± 0.038	0.840 ± 0.085	0.807 ± 0.088	0.363 ± 0.008	0.803 ± 0.032
Yea	0.291 ± 0.055	0.681 ± 0.031	0.461 ± 0.028	0.679 ± 0.034	**0.723** ± 0.012	**0.726** ± 0.013	0.233 ± 0.002	0.644 ± 0.049
Zoo	0.807 ± 0.041	0.983 ± 0.012	0.849 ± 0.047	**0.992** ± 0.016	0.928 ± 0.046	0.914 ± 0.039	0.753 ± 0.122	0.981 ± 0.010
**Mean**	0.565 ± 0.047	0.719 ± 0.039	0.614 ± 0.055	**0.748** ± 0.046	0.739 ± 0.046	0.730 ± 0.053	0.533 ± 0.027	0.728 ± 0.038
